# Influence of gender in monocrotaline and chronic hypoxia induced pulmonary hypertension in obese rats and mice

**DOI:** 10.1186/s12931-020-01394-0

**Published:** 2020-06-03

**Authors:** Balram Neupane, Akylbek Sydykov, Kabita Pradhan, Christina Vroom, Christiane Herden, Srikanth Karnati, Hossein Ardeschir Ghofrani, Sergey Avdeev, Süleyman Ergün, Ralph Theo Schermuly, Djuro Kosanovic

**Affiliations:** 1 Universities of Giessen and Marburg Lung Center (UGMLC), Member of the German Center for Lung Research (DZL), Aulweg 130, 35392 Giessen, Germany; 2 Medizinischen Klinik I, Universitätsklinikum RWTH Aachen, Pauwelsstraße 30, 52074 Aachen, Germany; 3 Institute of Veterinary Pathology, Justus-Liebig University, Giessen, Germany; 4 Institute of Anatomy and Cell Biology, Julius-Maximilians-University Würzburg, Würzburg, Germany; 5 Sechenov First Moscow State Medical University (Sechenov University), Moscow, Russia

**Keywords:** Obesity, Pulmonary hypertension, Zucker rats, Obese B6 mice, Monocrotaline, Inflammation, Hypoxia

## Abstract

**Background:**

Obesity and pulmonary hypertension (PH) share common characteristics, such as augmented inflammation and oxidative stress. However, the exact role of obesity in the pathology of PH is largely uninvestigated. Therefore, we have hypothesized that in the context of obesity the gender difference may have influence on development of PH in animal models of this disease.

**Methods:**

Animal experiments were conducted in monocrotaline (MCT) and chronic hypoxia (HOX) models of PH. Lean and obese Zucker rats or B6 mice of both genders were used for MCT or HOX models, respectively. Echocardiography, hemodynamic measurements, histology and immuno-histochemistry were performed to analyze various parameters, such as right ventricular function and hypertrophy, hemodynamics, pulmonary vascular remodeling and lung inflammation.

**Results:**

Both lean and obese male and female Zucker rats developed PH after a single MCT injection. However, negligible differences were seen between lean and obese male rats in terms of PH severity at the end stage of disease. Conversely, a more prominent and severe PH was observed in obese female rats compared to their lean counterparts. In contrast, HOX induced PH in lean and obese, male and female mice did not show any apparent differences.

**Conclusion:**

Gender influences PH severity in obese MCT-injected rats. It is also an important factor associated with altered inflammation. However, further research is necessary to investigate and reveal the underlying mechanisms.

## Background

Pulmonary hypertension (PH) is a disease characterized by increased pulmonary vascular resistance, alteration of the normal vascular cellular processes and inflammation in the lungs, which leads to right heart failure and death [1]. Obesity, a condition with the excessive adipose tissue accumulation in the body, has been previously shown to be associated with cardiovascular and pulmonary diseases. Obesity and obesity related complications are regarded to have impact on developing PH and early mortality [2, 3]. However, no clear data exists in connection to PH and obesity [4, 5]. Interestingly, conflicting effects of obesity were seen in patients diagnosed with pulmonary arterial hypertension (PAH) after first or third year of PAH diagnosis. The mortality in the first year after PAH diagnosis was higher, while it was significantly lower in obese patients after third year of diagnosis [6]. The role of obesity in PH is still unclear suggesting it neither as a friend nor as a foe. Systematic research, therefore, is needed to establish an apparent association between these two conditions.

Cardiac dysfunction and pulmonary vascular remodeling in PH have also been shown to be affected by gender and female sex hormones [7, 8]. Previously known to be predominantly present in younger females few decades ago, PAH recently has an even occurrence ratio in males and females [9]. Interestingly, female survival rate after the first diagnosis of PH is higher than in males [10]. Similarly, pre-clinical studies in rats with monocrotaline induced PH have shown less severe condition in female rats which has been attributed to their higher antioxidant defense capacity compared to male [8]. A recent study shows better long-term survival rate in females who have chronic thromboembolic PH, albeit the short term survival rates were identical in both genders [11]. This noticeable heterogeneity of females over males also requires further research to explore the gender specific alteration concerning obesity and PH.

Furthermore, a broad spectrum of inflammatory mediators and oxidative stress plays a critical role in both pre-clinical and clinical forms of PH [12, 13]. Similarly, obesity is responsible to trigger inflammation and oxidative stress leading to increased endothelial dysfunction and contributing to cardiovascular disease [14]. This suggests that a possible intervention on inflammatory pathway might be an effective future target for the treatment of PH.

Up-to-date, research evidences are not conclusive enough to understand the mechanisms and relationship between obesity and PH. This study addresses both the relation between obesity and PH and the differences between male and female obesity associated with PH. We have used well-established mouse and rat models of PH with both genders.

## Methods

### Animal models

Adult obese and lean Zucker rats (crl: ZUC-Lepr^fa^) (Charles River Laboratories, Sulzfeld, Germany) of both genders were maintained under controlled conditions, daylight/night cycle of 14/10 h and 22 ± 2 °C temperature with ad libitum food and water supply. Similarly, adult male and female lean (C57BL6/J or B6) and obese mice (ob/ob B6.Cg-Lep^ob^/J) (Charles River Laboratories, Sulzfeld, Germany) were also maintained under identical conditions as for the Zucker rats. The study protocols were approved by the governmental Animal Ethics Committee: Regierungspraesidium Giessen, GI 20/10 Nr. 24/2014 and GI 20/10 Nr. 71/2012.

### Study design

Part 1: Lean/obese Zucker rats, both males and females were injected with either monocrotaline (MCT) (60 mg/kg body weight; prepared using the HCL and NaOH) or normal saline subcutaneously (s/c). General body conditions and weight changes were monitored daily until 5 weeks. Echocardiography and hemodynamics measurements were performed 5 weeks after the MCT injection.

Part 2: Lean/obese B6 mice male and female were exposed to either hypoxia (10% O_2_) as described previously [15] or normoxia (21% O_2_) acting as control mice. Echocardiography and hemodynamic measurements were performed 5 weeks after the hypoxic or normoxic condition.

### Body mass index (BMI)

BMI was calculated for rats and mice of both genders by using the following formula: BMI = weight in grams/length in centimeters^2^. The length was measured from the tip of the nose until the base of the tail.

### Echocardiography

Echocardiography was performed as reported previously [16]. In general, the images were obtained with a VEVO2100 high resolution imaging system (Visual Sonics, Toronto, Canada) equipped with transducers MS550D (22–55 MHz) and MS250 (13–24 MHz). Right ventricular wall thickness (RVWT), right ventricular internal diameter (RVID) and tricuspid annular plane systolic excursion (TAPSE), as well as pulmonary artery acceleration time (PAAT) and pulmonary artery ejection time (PAET) were assessed.

### Hemodynamic measurements and right ventricular hypertrophy

The invasive hemodynamic catheterization was described previously [16]. Briefly, systemic arterial pressure (SAP) was measured from left carotid artery and right ventricular systolic pressure (RVSP) was measured in the right ventricle. As for the right ventricular hypertrophy (Fulton index), the heart was excised to right ventricle and left ventricle + septum and weight was taken.

### Histology and pulmonary vascular morphometry

Initially, the lungs were flushed through the pulmonary artery with 0.9% normal saline. The left lung was formalin fixed and paraffin embedded for histological analysis. For assessment of the pulmonary vascular remodeling (degree of muscularization and medial wall thickness) the protocols were followed as described previously [15, 16]. Briefly, the lungs were stained with Elastica van Gieson and the distance between *lamina elastica interna* and *externa* represents the thickness of the media. Light microscopy with a computer software for morphometry (Qwin, Leica, Germany) was engaged to measure the percentage of medial wall thickness using the following formula: medial wall thickness (%) = (2 x wall thickness/external diameter) × 100). For the degree of muscularization, the lungs were initially immunostained with anti-alpha smooth muscle actin antibody and anti-von-Willebrand factor antibody. Using the light microscopy and a special software for morphometry (Qwin, Leica, Germany) the percentage of fully muscularized vessels was calculated against the total number of counted vessels. All analyses were performed in a blinded manner.

### Immunohistochemistry and morphometric assessment of the lung inflammation

To quantify macrophages, CD68 staining was done in the lung tissues. The anti-Rat CD68 (MCA341R, AbDSerotec) antibody was used.

### Statistical analysis

All the values were expressed as mean ± standard error of mean (SEM). Experimental groups were compared by two-way ANOVA with Sidak’s multiple comparisons test. A *p* value of less the 0.05 was considered significant.

## Results

### Body mass index (BMI) and echocardiographic parameters in male Zucker rats

**Fig. 1:**
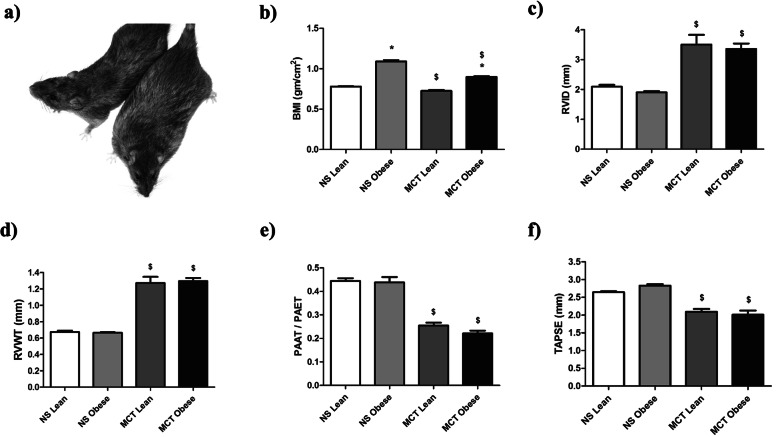
Effects of obesity on echocardiographic parameters in monocrotaline (MCT)-induced pulmonary hypertension (PH) in male Zucker rats. Echocardiography was performed after 5 weeks of either normal saline (NS) (NS Lean (*n* = 10); NS Obese (*n* = 9–10)) or monocrotaline (MCT Lean (*n* = 11); MCT Obese (*n* = 8–10)) treatment in lean and obese male Zucker rats. **a** Lean and obese male Zucker rats are shown. **b** Body mass index (BMI) of lean and obese male Zucker rats and (**c-f**) different echocardiographic parameters are given. RVID = right ventricular internal diameter, RVWT = right ventricular wall thickness, PAAT = pulmonary artery acceleration time, PAET = pulmonary artery ejection time, TAPSE = tricuspid annular plane systolic excursion. Data are presented as mean ± SEM (*n* = 8–11). *p* < 0.05 values are considered statistically significant. *compared to lean, ^$^compared to normal saline

Five weeks after the saline or MCT injection, there was a significant increase in BMI in obese male rats compared to their respective lean controls (Fig. 1a and b). Interestingly, there was a reduction of BMI in both lean and obese male rats upon the MCT application. As already mentioned, echocardiographic assessment of heart function was performed. Following MCT injection, both lean and obese male rats developed comparable PH, as evident by the increased values of RVID and RVWT, and decreased values of PAAT/PAET and TAPSE, in comparison to their respective normal saline controls (Fig. 1c-f).

### Hemodynamics and pulmonary vascular remodeling in male Zucker rats

**Fig. 2:**
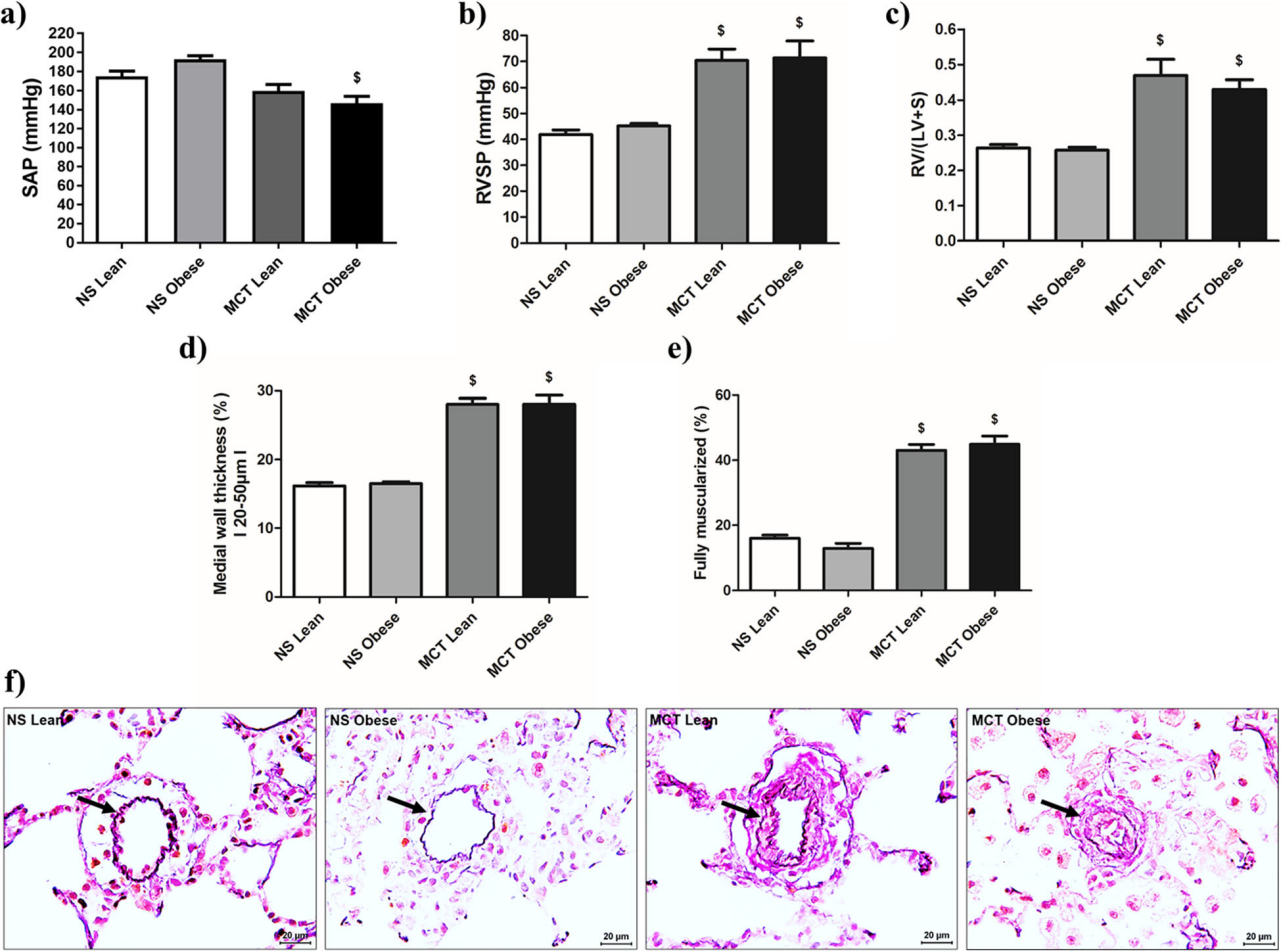
Effects of obesity on hemodynamics, right ventricular hypertrophy and pulmonary vascular remodeling in monocrotaline (MCT)-induced pulmonary hypertension (PH) in male Zucker rats. Hemodynamics and right ventricular hypertrophy measurements were performed after 5 weeks of either normal saline (NS) (NS Lean (n = 9–10); NS Obese (*n* = 9–10)) or monocrotaline (MCT Lean (*n* = 6–11); MCT Obese (*n* = 2–11)) treatment in lean and obese male Zucker rats. **a**, **b** Hemodynamic measurements and (**c**) Fulton’s index (weight ratio of right ventricle (RV) to left ventricle and septum (LV + S)) are shown. **d**-**e** Medial wall thickness and degree of muscularization are given. **f** Representative photomicrographs of medial wall thickness for different groups are depicted. Pulmonary vessels are indicated by arrows. SAP = systemic arterial pressure, RVSP = right ventricular systolic pressure. Data are presented as mean ± SEM (n = 2–11). *p* < 0.05 values are considered statistically significant. ^$^compared to normal saline

Invasive hemodynamics were performed after 5 weeks of normal saline or MCT injection. SAP was similar among the groups, except there was a decrease in MCT obese rats compared to their normal saline controls (Fig. 2a). Significant increase of RVSP shows PH in the MCT lean and obese Zucker rats (Fig. 2b). There was also significant increase in Fulton’s index in MCT lean and obese Zucker rats (Fig. 2c). In MCT injected lean and obese rats, there was increased medial wall thickness and fully muscularized vessels (Fig. 2d-e). However, no differences between the lean and obese male rats were seen.

### Body mass index (BMI) and echocardiographic parameters in female Zucker rats

**Fig. 3:**
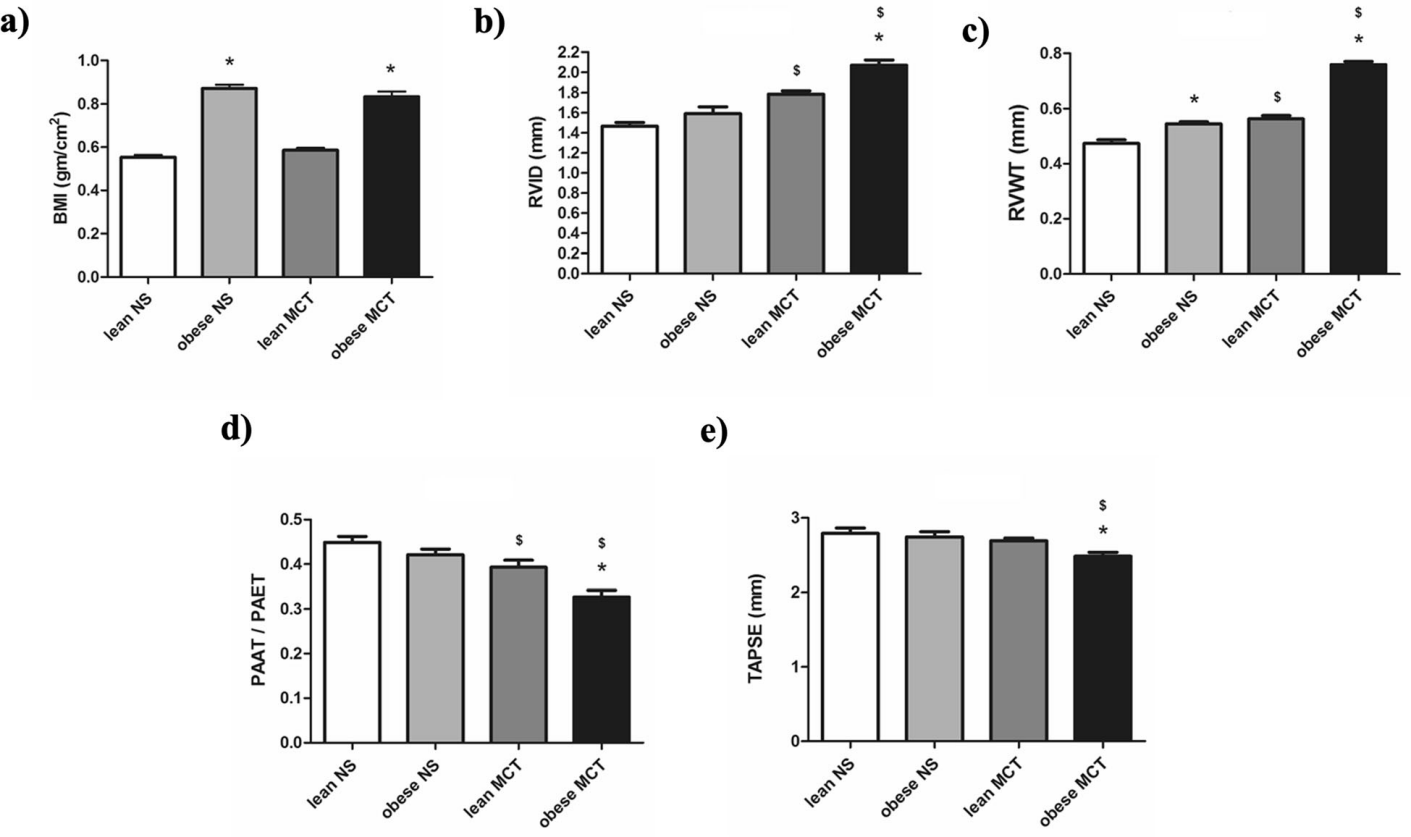
Effects of obesity on echocardiographic parameters in monocrotaline (MCT)-induced pulmonary hypertension (PH) in female Zucker rats. Echocardiography was performed after 5 weeks of either normal saline (NS) (lean NS (*n* = 10); obese NS (*n* = 10)) or monocrotaline (lean MCT (*n* = 10); obese MCT (*n* = 10)) treatment in lean and obese female Zucker rats. **a** Body mass index (BMI) of lean and obese female Zucker rats and (**b-e**) different echocardiographic parameters are given. RVID = right ventricular internal diameter, RVWT = right ventricular wall thickness, PAAT = pulmonary artery acceleration time, PAET = pulmonary artery ejection time, TAPSE = tricuspid annular plane systolic excursion. Data are presented as mean ± SEM (*n* = 10). *p* < 0.05 values are considered statistically significant. *compared to lean, ^$^compared to normal saline

BMIs of obese female rats were significantly higher than their lean counterparts taken after 5 weeks (Fig. 3a). In addition, there was no change of BMI in both lean and obese female rats upon the MCT application. The echocardiography performed after 5 weeks of normal saline or MCT injection showed that RVID and RVWT were more severe in the MCT injected obese female Zucker rats compared to the lean group (Fig. 3b-c). In addition, PAAT/PAET was reduced in MCT injected female Zucker rats in comparison to the saline controls (Fig. 3d). This was even more decreased in obese MCT rats compared to the lean counterparts. Finally, TAPSE was significantly reduced in obese female MCT rats compared to the lean group (Fig. 3e).

### Hemodynamics and pulmonary vascular remodeling in female Zucker rats

**Fig. 4:**
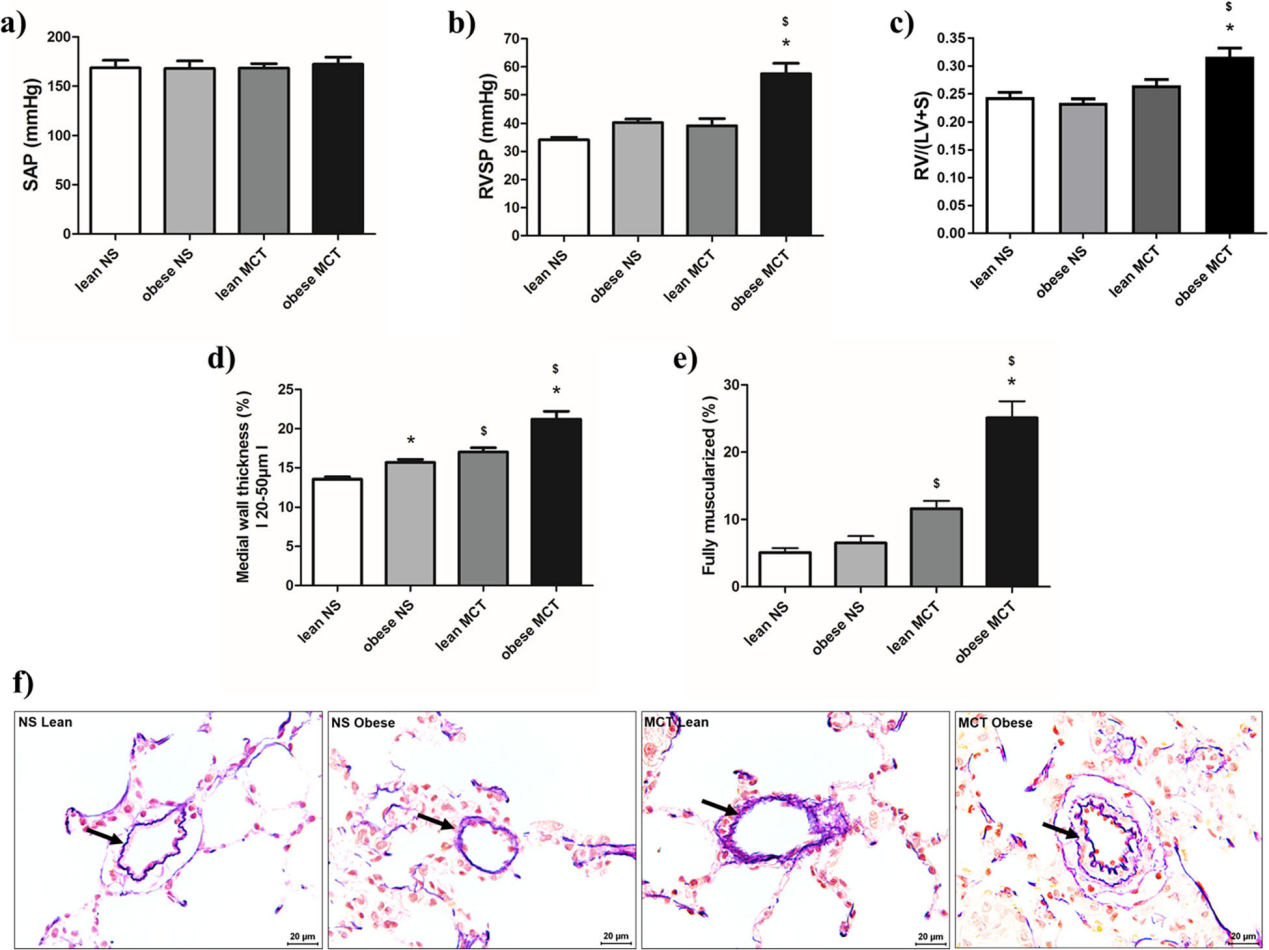
Effects of obesity on hemodynamics, right ventricular hypertrophy and pulmonary vascular remodeling in monocrotaline (MCT)-induced pulmonary hypertension (PH) in female Zucker rats. Hemodynamics and right ventricular hypertrophy measurements were performed after 5 weeks of either normal saline (NS) (lean NS (*n* = 10); obese NS (*n* = 10)) or monocrotaline (lean MCT (*n* = 10); obese MCT (*n* = 10)) treatment in lean and obese female Zucker rats. **a**, **b** Hemodynamic measurements and (**c**) Fulton’s index (weight ratio of right ventricle (RV) to left ventricle and septum (LV + S)) are shown. **d-e** Medial wall thickness and degree of muscularization are given. **f** Representative photomicrographs of medial wall thickness for different groups are depicted. Pulmonary vessels are indicated by arrows. SAP = systemic arterial pressure, RVSP = right ventricular systolic pressure. Data are presented as mean ± SEM (*n* = 10). *p* < 0.05 values are considered statistically significant. *compared to lean, ^$^compared to normal saline

Hemodynamics performed after 5 weeks of normal saline or MCT injection showed no differences in SAP (Fig. 4a), while there was a significant increase in RVSP and Fulton’s index in MCT injected obese female Zucker rats compared to other relevant groups (Fig. 4b-c). The lean group remained less affected by MCT injection. Corresponding to RVSP and Fulton’s index in MCT injected female Zucker rats, there were increased medial wall thickness and fully muscularized vessels as compared to the respective saline groups (Fig. 4d-e). In addition, the pulmonary vascular remodeling was more severe in MCT injected obese female rats, in comparison to the lean counterparts (Fig. 4d-e). Interestingly, the normal saline treated obese group had increased medial wall thickness compared to lean group (Fig. 4d).

### Lung inflammation in male and female Zucker rats

**Fig. 5:**
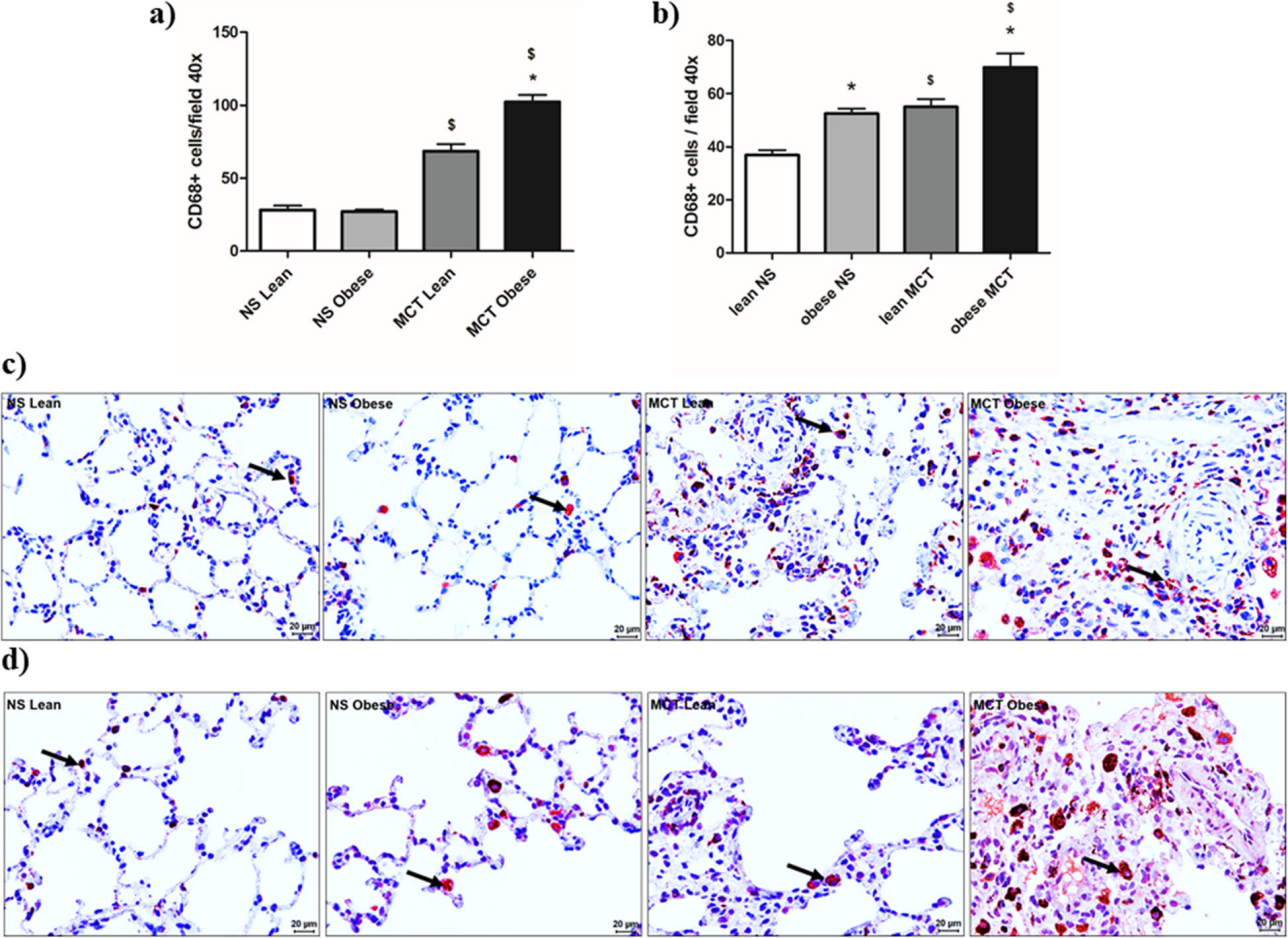
Effects of obesity on pulmonary inflammation in monocrotaline (MCT)-induced pulmonary hypertension (PH) in male and female Zucker rats. The quantification and representative photomicrographs of CD68 (macrophages) positive cells in the lung tissues of (**a, c**) male (*n* = 6–11) and (**b, d**) female (*n* = 8–10) Zucker rats are given. Arrows indicate the CD68 positive cells. NS = normal saline. Data are presented as mean ± SEM (*n* = 6–11). *p* < 0.05 values are considered statistically significant. *compared to lean, ^$^compared to normal saline

In male Zucker rats, higher infiltration of CD68-positive cells was detected in MCT- injected lean and obese groups and it was further increased in the obese MCT group compared to the lean MCT group (Fig. 5a and c). In female Zucker rats, high infiltrations of CD68-positive cells were detected in MCT injected lean and obese groups, in comparison to the respective normal saline controls (Fig. 5b and d). Obesity induced a significant increase in CD68-positive cells in both saline and MCT treated female Zucker rats compared to the lean groups.

### Effect of chronic hypoxia in B6 male mice

**Fig. 6:**
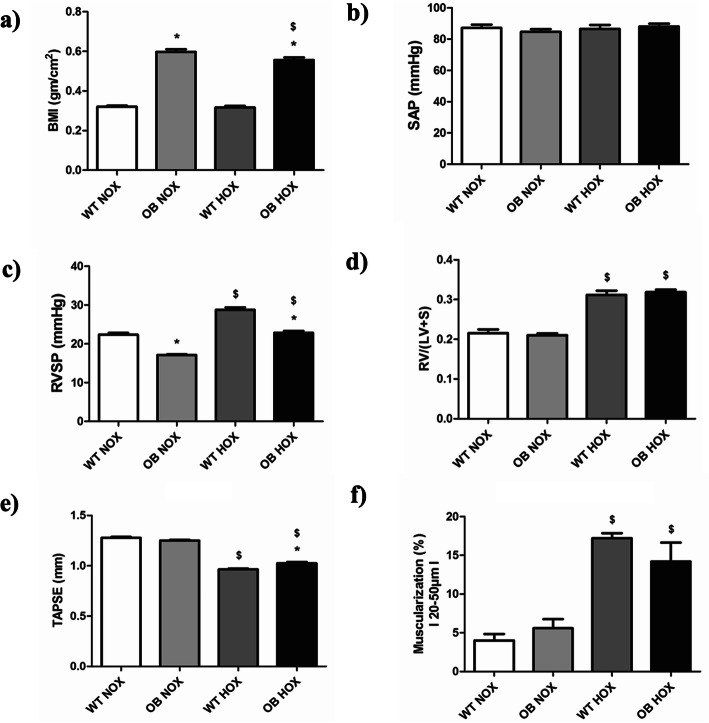
Effects of obesity on hemodynamics, right ventricular hypertrophy and function, and pulmonary vascular remodeling in chronic hypoxia (HOX)-induced pulmonary hypertension (PH) in male B6 mice. Hemodynamics and right ventricular hypertrophy measurements were performed after 5 weeks of either normoxic (NOX) (WT NOX (*n* = 5–10); OB NOX (*n* = 5–20)) or hypoxic (HOX) (WT HOX (*n* = 5–10); OB HOX (*n* = 5–20)) exposure in wild type (WT) lean and obese (OB) male B6 mice. **a** Body mass index (BMI) and (**b-e**) different hemodynamic and right ventricular hypertrophy/function parameters are given. **f** Degree of muscularization (fully muscularized pulmonary vessels) is shown. SAP = systemic arterial pressure, RVSP = right ventricular systolic pressure, RV = right ventricle, LV + S = left ventricle plus septum, TAPSE = tricuspid annular plane systolic excursion. Data are presented as mean ± SEM (*n* = 5–20). *p* < 0.05 values are considered statistically significant. *compared to wild type, ^$^compared to normoxia

Mice kept under normoxic or hypoxic conditions were measured for BMI, echocardiographic parameters and hemodynamic parameters after 35 days. The obese male mice had significantly higher BMI than their lean counterparts (Fig. 6a). Echocardiography and hemodynamic parameters were taken 5 weeks after normoxia or hypoxia exposures. SAP was constant in all groups (Fig. 6b). TAPSE was decreased in hypoxic groups compared to their normoxic controls and there was slight increase of TAPSE in obese male mice in comparison to their lean counterparts (Fig. 6e). RVID was increased in hypoxic groups compared to their normoxic controls and there was a reduction of this parameter in obese male mice in comparison to their lean counterparts (Supplementary figure 1a). RVWT was also increased in hypoxic groups compared to their normoxic controls, but conversely to RVID, there was an elevation of this parameter in obese male mice in comparison to their lean counterparts (Supplementary figure 1b). Furthermore, PAAT/PAET was reduced in male mice exposed to hypoxia and this parameter was slightly increased in obese mice compared to their lean counterparts under both normoxic and hypoxic conditions (Supplementary figure 1c). Invasive hemodynamics done after 5 weeks of normoxia or hypoxia exposure showed lower RVSP for both normoxic and hypoxic obese male mice and higher RVSP due to hypoxia was observed (Fig. 6c). The Fulton’s index was higher in lean and obese hypoxic groups compared to the respective normoxia controls (Fig. 6d). Pulmonary vascular remodeling (fully muscularized pulmonary vessels) was more prominent in both lean and obese hypoxic groups in comparison to the respective normoxic conditions (Fig. 6f). In addition, medial wall thickness was increased in lean male mice exposed to hypoxia compared to the normoxic control (Supplementary figure 1d). However, there was no change of this parameter in the case of obese male mice under hypoxic condition (Supplementary figure 1d).

### Effect of chronic hypoxia in B6 female mice

**Fig. 7:**
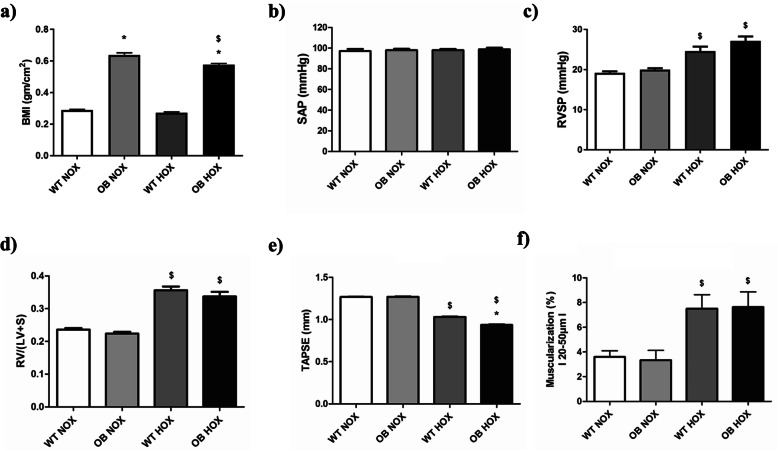
Effects of obesity on hemodynamics, right ventricular hypertrophy and function, and pulmonary vascular remodeling in chronic hypoxia (HOX)-induced pulmonary hypertension (PH) in female B6 mice. Hemodynamics and right ventricular hypertrophy measurements were performed after 5 weeks of either normoxic (NOX) (WT NOX (*n* = 10); OB NOX (*n* = 9–10)) or hypoxic (HOX) (WT HOX (*n* = 10); OB HOX (*n* = 8–10)) exposure in wild type (WT) lean and obese (OB) female B6 mice. **a** Body mass index (BMI) and (**b-e**) different hemodynamic and right ventricular hypertrophy/function parameters are given. **f** Degree of muscularization (fully muscularized pulmonary vessels) is shown. SAP = systemic arterial pressure, RVSP = right ventricular systolic pressure, RV = right ventricle, LV + S = left ventricle plus septum, TAPSE = tricuspid annular plane systolic excursion. Data are presented as mean ± SEM (*n* = 8–10). *p* < 0.05 values are considered statistically significant. *compared to wild type, ^$^compared to normoxia

The obese female mice had significantly higher BMIs than their lean counterparts (Fig. 7a). SAP was constant in all groups (Fig. 7b). TAPSE was decreased in hypoxic groups compared to their normoxic controls and slight reduction of TAPSE was seen in obese female hypoxic mice in comparison to their lean counterparts (Fig. 7e). RVID was increased in hypoxic groups compared to their normoxic controls and there was a further elevation of this parameter in obese female mice in comparison to their lean counterparts (Supplementary figure 2a). RVWT was also increased in hypoxic groups compared to their normoxic controls (Supplementary figure 2b). In addition, there was slight increase of this parameter in obese female mice in comparison to their lean counterparts under both normoxia and hypoxia (Supplementary figure 2b). Furthermore, PAAT/PAET was reduced in female mice exposed to hypoxia and this parameter was slightly decreased in obese mice compared to their lean counterparts under normoxic conditions (Supplementary figure 2c). Hypoxic female mice had higher RVSP and Fulton’s index compared to the respective normoxic groups, and there was no influence of obesity (Fig. 7c-d). Fully muscularized vessels were increased in hypoxic lean and obese female mice, in comparison to their normoxic controls (Fig. 7f). Finally, medial wall thickness was increased in lean female mice exposed to hypoxia compared to the normoxic control (Supplementary figure 2d). However, there was no change of this parameter in the case of obese female mice under hypoxic condition (Supplementary figure 2d).

## Discussion

Globally, around 107.7 million children and 603.7 million adults were obese in 2015 with a prevalence of 5 and 12% in children and adults, respectively [17]. Though the data are insufficient about the prevalence of PH in obese people, it has been shown that 5% of people with body mass index (BMI) > 30 kg/m^2^ had PH with pulmonary artery systolic pressure (PASP) > 40 mmHg, as measured echocardiographically [18]. In addition Wong et al. showed the increased severity of right ventricular (RV) dysfunction with increasing BMI [19]. Although obesity is a growing worldwide problem and is associated with many diseases, there is an ongoing debate if some underlying factors of obesity have beneficial effect in heart disease.

Obesity is featured by excessive adipose tissue accumulation in the body. These adipose tissues release adipokines [20, 21]. Recently, it has been shown that there is a relation between adipokine dysregulation and hemodynamic disorders in PAH [22]. Obesity is associated with low mortality in some PH patients indicating it might have a protective effect [23]. Diong et al. have demonstrated that sympathetic nerve activity which facilitates the pulmonary vasodilatation was increased in obesity or chronic hypoxia. This hyperactivity may help to decrease the severity of PH [24].

The role of obesity is obviously still unclear in relation to PH. A retrospective case control study regarding the association between obesity and PH did not show any correlations with class I obesity. However, there was a slight indication of possible connection with class II or class III obesity [25]. Animal experiments in relation to gender have shown that estrogens have protective role in animal models of PH [26].

Mild increment of pulmonary arterial pressure, RV hypertrophy and pulmonary artery thickening have been shown in Zucker diabetic fatty rats [27]. Irwin and coworkers demonstrated the development of PH in chow-fed Zucker rats. Adipose tissue and liver acted as a source for circulating free fatty acids and triglycerides respectively [4]. When the clearance of lipoproteins by the liver is hindered, there is increase in fat delivery to the pulmonary arterial wall affecting vascular structure and function. The inefficient oxidation of fatty acids results in the production of reactive oxygen species [28, 29]. Likewise Irwin et al. also showed that PH in Zucker rats showed no inflammatory cytokines but rather the obese rat model with high fat diet showed the inflammatory cytokines increment [4]. However, in the present study, we used MCT to induce PH and demonstrated that obesity did not influence the disease severity in MCT-induced PH in male Zucker rats. In details, our data revealed that the parameters of right ventricular hypertrophy/remodeling (RVID, RVWT and RV/(LV + S)) and function (TAPSE) were comparable between male MCT obese Zucker rats and their lean controls. We have shown previously that PAAT was reduced in MCT rat model and this parameter negatively correlated with RVSP [16]. In the present study, obesity did not change the value of PAAT/PAET in MCT treated male rats. Similarly, there was no influence of obesity in male MCT rats with regard to the pulmonary vascular remodeling, which represents the hallmark of PH pathology, as evident from the medial wall thickness and muscularization measurements. Finally, our hemodynamic data indicated that obesity did not affect one of the most important characteristic features of PH, such as RVSP, in MCT treated animals. In addition, there were no prominent changes in SAP due to obesity. There was only slight reduction of SAP in MCT obese rats in comparison to their saline control, and this might appear because MCT injected animals develop a severe pulmonary vascular disease. However, after MCT injection there was more prominent inflammation (CD68-positive cells) present in the lungs of obese male rats compared to the lean counterparts. Inflammatory cytokines and chemokines in PAH are important attributes of PH. In obese individuals, the cytokines produced by the adipose tissues might add-up to the already present inflammation in the lung tissues aggravating pulmonary vascular pathology [4, 30].

Female obese Zucker rats, on the other hand, showed high severity of the disease but less effects of MCT-induced PH. In details, our data demonstrated that obesity prominently worsened the right ventricular hypertrophy/remodeling (RVID, RVWT and RV/(LV + S)) and function (TAPSE) in female MCT Zucker rats in comparison to their lean controls. Furthermore, obesity reduced the value of PAAT/PAET and significantly enhanced the pulmonary vascular remodeling in MCT treated female rats. Importantly, one of the main characteristics of PH, such as elevated RVSP, was noticeably increased in obese female MCT rats compared to their lean counterparts. Finally, there were no alterations in SAP among the experimental groups. The difference of MCT-induced PH in male and female rats have been shown to be due to female hormones and oxidative stress [8, 31]. Our results indicate that obesity might represent an important factor modulating the PH development in the context of females. Therefore, obese Zucker female rats are more susceptible to pulmonary vascular remodeling and right ventricular dysfunction after MCT injection. As in males, upon the MCT injection there was increased accumulation of CD68-positive cells in the lungs of obese female rats compared to the lean counterparts. In contrast to the males, the female obese rats injected with saline were characterized by augmented number of CD68-positive cells in comparison to their lean controls.

Interestingly, there was a significant reduction of BMI in male Zucker rats upon the induction of PH with MCT. This phenomenon was not observed in the context of female Zucker rats. In general, the weight loss may appear due to harsh pulmonary vascular disease in MCT model, and such discrepancy may be attributed to already mentioned fact that MCT caused more severe disease in male rats as compared to the females.

The REVEAL and COMPERA registry have shown that PAH predominantly affects females [9, 32]. Therefore, the hormone might have influence on PAH development. However, irrespective to the disease severity, the prevalence of male mortality with PAH was twice as compared to females [32, 33].

Chronic hypoxia induced model in mice is used as a PH model in several studies [34, 35]. Although some data with regard to the right ventricular remodeling and function (RVID and TAPSE), PAAT/PAET and medial wall thickness showed a mild “improvements” of these PH parameters in obese versus lean male mice under hypoxia, other important measures demonstrated either a slight worsening of the right ventricular hypertrophy (RVWT) or no change (RV/(LV + S)). In addition, there were no significant differences in the muscularization between obese and lean male mice exposed to hypoxia. Furthermore, invasive hemodynamic measurement of RVSP, as one of the main feature of PH, revealed the increase of this parameter due to hypoxia exposure in both lean and obese male mice. Surprisingly, there were significant differences under the baseline conditions and reduction of RVSP even in obese male mice under normoxia compared to their lean counterparts. In the case of female mice, some parameters indicated that right ventricular structure (RVID and RVWT) and function (TAPSE) were a bit worse in obese female mice in comparison to the lean controls under hypoxic conditions. Conversely, the medial wall thickness was reduced, while there were no differences in the muscularization between obese and lean female mice exposed to hypoxia. Importantly, the main PH parameters derived from the invasive measurements of the hemodynamics and right ventricular hypertrophy, such as RVSP and Fulton’s index, did not show any effect of obesity in female mice in chronic hypoxia-induced PH. In both male and female mice, there was no change of SAP among the experimental groups. Overall, in the case of hypoxia-induced PH, obesity was not convincingly demonstrated to play an important role in both genders. In contrast, it was shown that mild form of PH is higher in obese patients living at higher altitude with low oxygen [36]. Therefore, the future studies are crucially needed for final establishment of whether or not obesity modifies chronic hypoxia-induced PH.

Both obese male and obese female Zucker rats showed higher degree of inflammation (CD68-positive cells) in the lung tissues following MCT injection. Lungs from both idiopathic PAH patients as well as MCT-injected rats, have higher infiltration of peri-vascular inflammatory cells [37]. Our studies contribute to the fact that obesity accelerates the degree of inflammation and suggests that obesity might have crucial inflammatory effects to add up the disease severity.

## Conclusions

Obesity and PH are characterized by increased oxidative stress and inflammation. Though many studies have shown the possible correlation between obesity and PH, the exact pathology is not known yet. This study provides a clear role of inflammation in the MCT model of PH in obese Zucker rats with significant differences between genders. Further studies are required to substantiate these findings.

## Supplementary information

**Additional file 1: Figure S1.** Effects of obesity on echocardiographic parameters and pulmonary vascular remodeling in chronic hypoxia (HOX)-induced pulmonary hypertension (PH) in male B6 mice. **Figure S2.** Effects of obesity on echocardiographic parameters and pulmonary vascular remodeling in chronic hypoxia (HOX)-induced pulmonary hypertension (PH) in female B6 mice.

## Data Availability

All data are available from the corresponding author on reasonable request.

## Abbreviations

HOX: Chronic hypoxia
MCT: Monocrotaline
PH: Pulmonary hypertension
PAH: Pulmonary arterial hypertension
BMI: Body mass index
SAP: Systemic arterial pressure
RVSP: Right ventricular systolic pressure
RVWT: Right ventricular wall thickness
RVID: Right ventricular internal diameter
TAPSE: Tricuspid annular plane systolic excursion
PAAT: Pulmonary artery acceleration time
PAET: Pulmonary artery ejection time
PASP: Pulmonary artery systolic pressure
