# Supplementary material for: Influence of gender in monocrotaline and chronic hypoxia induced pulmonary hypertension in obese rats and mice
Source: Respir Res. 2020 Jun 3;21:136. doi: 10.1186/s12931-020-01394-0 (PMC7268383; doi:10.1186/s12931-020-01394-0)
Supplement: Supplementary file 1 — Additional file 1: Figure S1. Effects of obesity on echocardiographic parameters and pulmonary vascular remodeling in chronic hypoxia (HOX)-induced pulmonary hypertension (PH) in male B6 mice. Figure S2. Effects of obesity on echocardiographic parameters and pulmonary vascular remodeling in chronic hypoxia (HOX)-induced pulmonary hypertension (PH) in female B6 mice. [file 12931_2020_1394_MOESM1_ESM.pdf]

Influence of gender in monocrotaline and chronic hypoxia induced pulmonary hypertension in obese rats and mice

Balram Neupane; Akylbek Sydykov; Kabita Pradhan; Christina Vroom; Christiane Herden; Srikanth Karnati; Hossein Ardeschir Ghofrani; Sergey Avdeev; Süleyman Ergün; Ralph Theo Schermuly; Djuro Kosanovic

ONLINE SUPPLEMENT MATERIAL

Results

Supplementary figure 1. Effects of obesity on echocardiographic parameters and pulmonary vascular remodeling in chronic hypoxia (HOX)-induced pulmonary hypertension (PH) in male B6 mice.

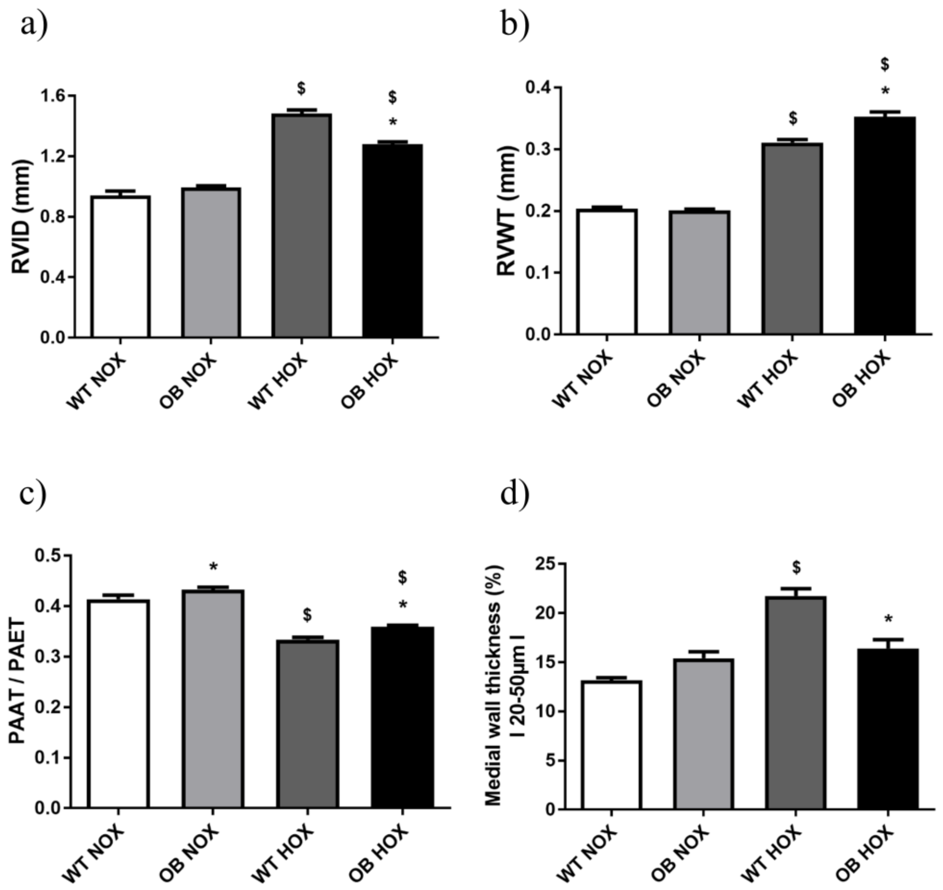

Echocardiography and medial wall thickness measurements were performed after 5 weeks of either normoxic (NOX) (WT NOX (n=5-10); OB NOX (n=5-20)) or hypoxic (HOX) (WT HOX (n=5-10); OB HOX (n=5-20)) exposure in wild type (WT) lean and obese (OB) male B6 mice. (**a-c**) Different echocardiographic parameters are given. RVID = right ventricular internal diameter, RVWT = right ventricular wall thickness,

PAAT = pulmonary artery acceleration time, PAET = pulmonary artery ejection time. (**d**) Medial wall thickness is shown. Data are presented as mean ± SEM (n=5-20). $p < 0.05$ values are considered statistically significant. *compared to wild type, $^{\$}$compared to normoxia.

Supplementary figure 2. Effects of obesity on echocardiographic parameters and pulmonary vascular remodeling in chronic hypoxia (HOX)-induced pulmonary hypertension (PH) in female B6 mice.

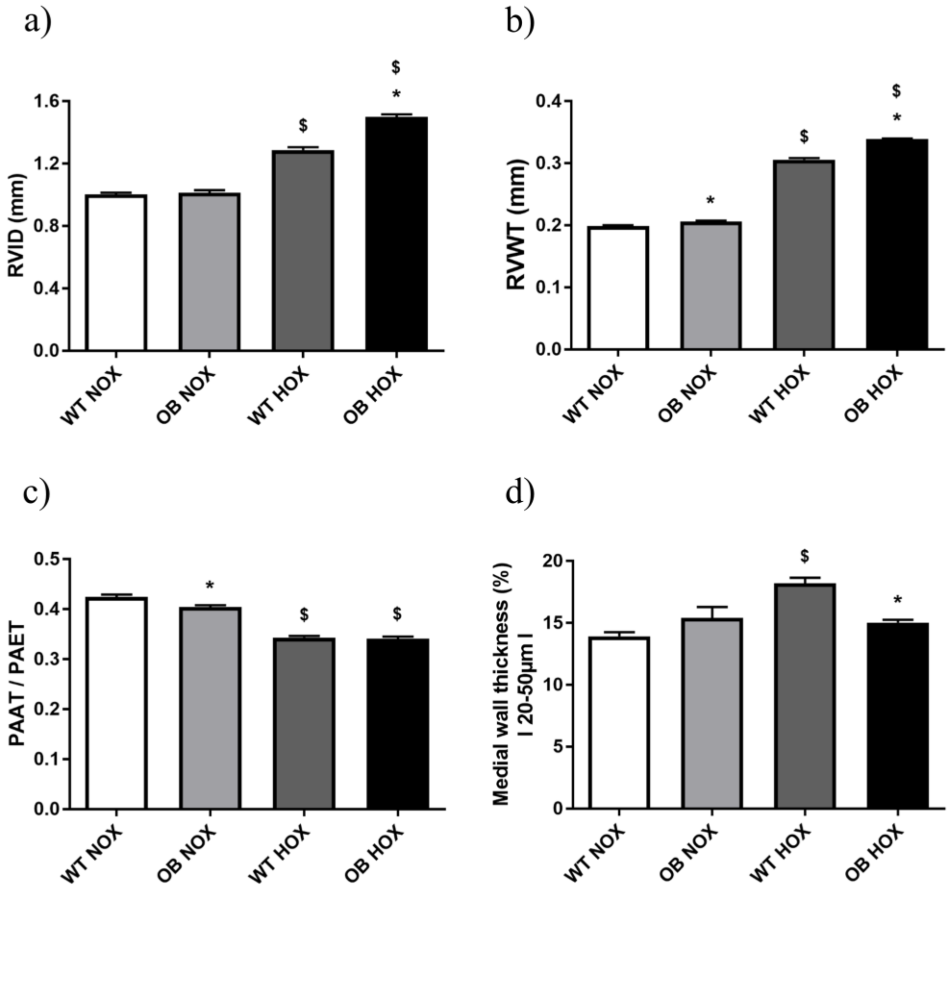

Echocardiography and medial wall thickness measurements were performed after 5 weeks of either normoxic (NOX) (WT NOX (n=5-10); OB NOX (n=5-10)) or hypoxic (HOX) (WT HOX (n=5-10); OB HOX (n=5-9)) exposure in wild type (WT) lean and obese (OB) female B6 mice. (**a-c**) Different echocardiographic parameters are given. RVID = right ventricular internal diameter, RVWT = right ventricular wall thickness, PAAT = pulmonary artery acceleration time, PAET = pulmonary artery ejection time. (**d**) Medial wall thickness is shown. Data are presented as mean ± SEM (n=5-10). $p < 0.05$ values are considered statistically significant. *compared to wild type, $^{\$}$compared to normoxia.
